# Physical exercise: a multi-target strategy for the prevention and treatment of Alzheimer’s disease

**DOI:** 10.3389/fnagi.2026.1876075

**Published:** 2026-10-19

**Authors:** Wuyun Tong

**Affiliations:** School of Physical Education, Nanchang University, Nanchang, China

**Keywords:** Alzheimer’s disease, Aβ, BDNF, cerebral metabolism, neuroinflammation, physical exercise, tau protein

## Abstract

Alzheimer’s disease (AD) is a devastating age-related neurodegenerative disease characterized by irreversible cognitive dysfunction and neuronal loss. The complex pathogenesis of AD involves core proteinopathies of amyloid-*β* (Aβ) and tau, chronic neuroinflammation, and extensive cerebral metabolic disorders, which collectively drive progressive neuronal damage and disease deterioration. Currently, effective disease-modifying drugs for AD are still lacking, and physical exercise has been recognized as a safe and effective non-pharmacological intervention to prevent and alleviate AD pathological progression. Specifically, exercise inhibits abnormal Aβ deposition and tau hyperphosphorylation, suppresses microglial overactivation and sustained neuroinflammatory responses, and remodels cerebral metabolic homeostasis by improving glucose utilization, balancing lactate metabolism, restoring mitochondrial function, and correcting dysregulated lipid metabolism. Additionally, exercise-mediated upregulation of brain-derived neurotrophic factor (BDNF) enhances neuronal survival and synaptic plasticity to block AD pathological progression. This review innovatively combines core AD pathological markers and multi-dimensional metabolic mechanisms, providing a theoretical reference for further exploring the molecular mechanism of exercise intervention in AD.

## 1 Introduction

Alzheimer’s disease (AD) is the most prevalent progressive neurodegenerative disorder and the leading cause of dementia worldwide, characterized by irreversible cognitive decline, memory impairment, and progressive neuronal loss that severely compromises the quality of life of elderly individuals and imposes a substantial socioeconomic burden on global healthcare systems (Courade et al., 2025). Regular physical exercise stands out as an accessible, low-risk non-pharmacological strategy for both preventing and managing AD (Chen et al., 2025; Zhang et al., 2022; Pitzalis et al., 2015).

AD is canonically defined by two pathological hallmarks: excessive amyloid-*β* (Aβ) plaques depositing in the extracellular brain parenchyma, and intracellular neurofibrillary tangles of hyperphosphorylated tau. Neuronal dysfunction and cognitive decline in AD stem from two parallel pathological processes: Aβ-driven neurotoxicity from dysregulated production, aggregation, and clearance, and tau-driven disruption of cytoskeletal stability, axonal transport, and synaptic integrity (Courade et al., 2025; Glenner and Wong, 1984; Hardy and Selkoe, 2002). Beyond these core proteinopathies, persistent neuroinflammation acts as a critical pathological amplifier in AD progression. Chronic overactivation of microglia and astrocytes induces the sustained release of pro-inflammatory cytokines, which exacerbate Aβ and tau pathology, amplify neuronal oxidative stress, and create a self-perpetuating neurotoxic microenvironment that accelerates neurodegeneration (Reddy and Oliver, 2019; Thakur et al., 2023; Long et al., 2025).

Cerebral metabolic dysfunction is an upstream and pivotal driver of AD pathogenesis, closely interacting with proteinopathy and neuroinflammation to exacerbate brain injury. Insulin resistance disrupts neuronal energy supply and compromises cognitive function (Winkler et al., 2015). Abnormal lactate accumulation further disturbs cerebral energy homeostasis (Zebhauser et al., 2022). Defective mitochondrial biogenesis, dynamics, and mitophagy drive metabolic failure, which in turn fuels ROS overproduction, oxidative injury, and neuronal apoptosis (Chan, 2006; Wang et al., 2022). In addition, dysregulated lipid metabolism in the brain impairs membrane integrity, lipid signaling, and Aβ clearance, further aggravating AD pathological progression (Hur, 2022; Wang et al., 2021).

Mounting evidence has validated that physical exercise exerts multi-target neuroprotective effects against AD by modulating the aforementioned pathological pathways. Nevertheless, the integrated regulatory mechanisms of exercise on the interactive crosstalk among Aβ/tau protein pathology, neuroinflammation, and metabolism in AD remain not fully elucidated. Therefore, this review summarizes the protective effects and molecular mechanisms of physical exercise against AD. This work aims to provide a theoretical basis for the optimization of exercise intervention strategies and the development of novel non-pharmacological therapeutic targets for Alzheimer’s disease (Figure 1).

**Figure 1:**
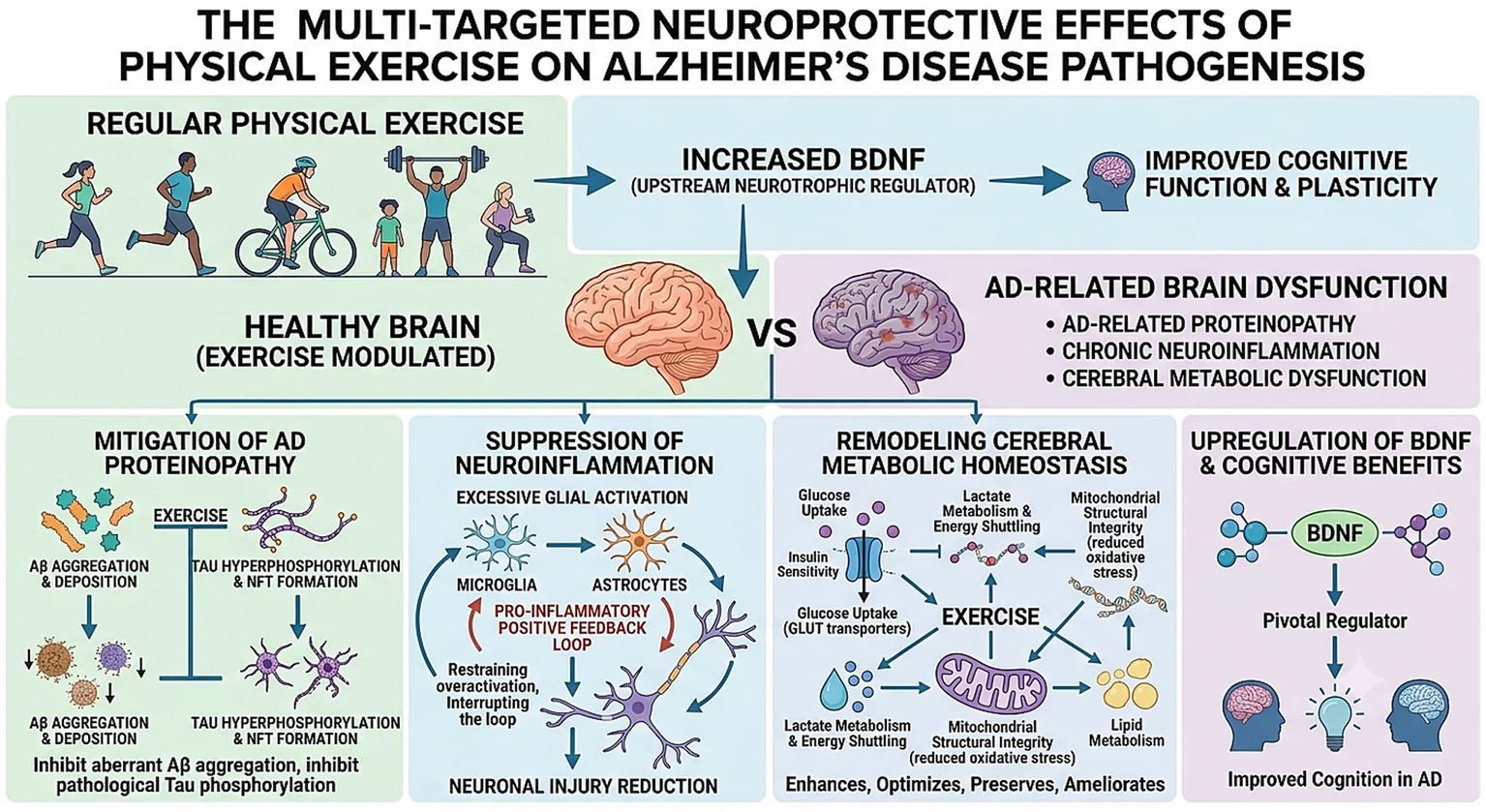
Multi-targeted neuroprotective mechanisms of physical exercise in Alzheimer’s disease pathogenesis. This schematic diagram illustrates how physical exercise acts as a potent, multi-targeted neuroprotective strategy to counteract the multifactorial pathogenesis of Alzheimer’s disease (AD) by simultaneously inhibiting aberrant Aβ aggregation and pathological Tau hyperphosphorylation to mitigate proteinopathy, restraining microglial and astrocytic overactivation to interrupt the pro-inflammatory neurodestructive feedback loop, comprehensively remodeling cerebral metabolic homeostasis via enhanced glucose uptake, optimized lactate shuttling, preserved mitochondrial integrity against oxidative stress, and ameliorated lipid metabolism, and ultimately upregulating BDNF as a pivotal upstream neurotrophic regulator to enhance synaptic plasticity and improve overall cognitive function.

## 2 Aβ and tau

The two principal neuropathological hallmarks of AD are protein-misfolding aggregates: Aβ plaques and tau tangles. One involves the extracellular deposition of amyloid-β (Aβ), resulting in the formation of amyloid plaques. While the other, intracellular accumulation of hyperphosphorylated tau (p-tau), leads to the development of neurofibrillary tangles (NFTs) (Courade et al., 2025; Hardy and Selkoe, 2002). These two disorders interact and ultimately result in neurodegeneration and clinical cognitive deterioration. In AD, Aβ-related abnormalities typically emerge earlier and progressively reshape a pro-pathological microenvironment through synaptotoxicity, glia-mediated neuroinflammatory responses, and cellular homeostatic stress. This change lowers the biological threshold for the initiation and expansion of tau pathology, promoting the propagation and accumulation of abnormal tau within neural circuits (Zhang et al., 2023). In contrast, the topography of tau pathology aligns more closely with neuronal injury and clinical symptoms, placing tau as a more proximal pathological correlate of neurodegeneration and cognitive decline. In other words, tau pathology may translate upstream Aβ perturbations and inflammation- or stress-related molecular events into cumulative structural damage and functional deterioration. The following sections summarize key mechanisms of Aβ and tau in AD pathogenesis.

### 2.1 Aβ and Alzheimer’s disease

Aβ is the main component of extracellular amyloid plaques in the brain of patients with AD and has long been considered a key molecular driver of the occurrence and progression of the disease (Glenner and Wong, 1984). The classical amyloid cascade hypothesis holds that the accumulation of Aβ is the initial pathological event, and then promotes tau protein aggregation, synapses, and neuronal dysfunction, and ultimately leads to cognitive decline (Hardy and Selkoe, 2002; Hardy and Higgins, 1992). Aβ is produced by cutting the amyloid precursor protein (APP) through the sequential cutting of β-secreting enzyme (BACE1) and *γ*-secreting enzyme (Deng et al., 2013). On the other hand, under physiological conditions, APP processing more commonly proceeds via the *α*-secretase–mediated non-amyloidogenic pathway, which precludes Aβ formation (Deng et al., 2013; Zhang et al., 2017). Accordingly, the central issue in AD is homeostasis between Aβ production and clearance (Bateman et al., 2006). Aβ clearance is mediated by multiple coordinated routes, such as enzymatic degradation, cerebrospinal/interstitial fluid drainage, and transport across the blood–brain barrier (BBB) (Engelhardt et al., 2016; Tarasoff-Conway et al., 2015). BBB efflux and influx involve several transport systems, including LRP1, ABCB1/ABCA1, and RAGE. In AD, reduced expression of efflux transporters together with upregulation of RAGE has been implicated in increasing the cerebral Aβ burden.

More and more evidence shows that exercise can reduce the accumulation of Aβ. Zhang et al. reported that in 13-month-old APP/PS1 mice, long-term autonomous wheel movement can improve learning and identification memory without affecting the exercise ability, reducing the Aβ plaque load of the hippocampal body, and increasing the volume of the dentate return and CA1 region. These changes are accompanied by the enhancement of hippocal glucose metabolism and changes in the metabolism and activation phenotype of microglia (GLUT5↑, CD68↓), while TREM2 shedding is inhibited (Zhang et al., 2022). In addition, Chen et al. found that in 6.5-month-old 5 × FAD mice, 1 month of treadmill training increased the tube diameter of the meningeal lymphatic vessels, enhanced its structural plasticity and drainage ability, and increased the transport of Aβ and glial fiber acid protein to deep cervical lymph nodes. It is related to the reduction of Aβ deposition in the hippomapa and frontal cortex, the proliferation of reactive glial cells and the reduction of astroglial cell aging, and the improvement of cognitive function (Chen et al., 2025). In summary, exercise may delay the occurrence of AD and slow down the progression of the disease by accelerating the removal of Aβ.

### 2.2 Tau and AD

Under physiological conditions, tau protein is a microtubule-related protein in the neuronal cytoplasm, which is responsible for regulating microtubule dynamics and supporting axon transport and nerve process growth. However, in AD, tau protein will undergo abnormal phosphorylation, conformational changes, and wrong folding, leading to dissociation from microtubules and forming soluble oligomers and insoluble aggregates, and finally depositing to form NFTs (Iqbal et al., 2016). Unlike the role of Aβ in the relatively early stage of AD, tau protein pathology is usually more closely related to the expression of neurodegeneration and clinical symptoms (Ossenkoppele et al., 2016). Spatial distribution of tau biomarkers and tau imaging is more consistent with cognitive disorder patterns and clinical staging, and can predict subsequent cognitive decline and brain atrophy (Meyer et al., 1986). In addition, pathological tau protein can be transmitted along anatomical and functional connections. This process is considered to involve the release of abnormal tau protein, intersynaptic transfer or ingestion through engulation, template-mediated intracellular aggregation, and subsequent regional expansion promoted by activity-dependent mechanisms (Mudher et al., 2017). In this sense, tau protein is not only a by-product of local deposition, but also a pathological entity that can self-amplify and reshape network functions in the brain. At the same time, the current diagnostic framework is increasingly emphasizing the characterization of potential disease processes based on biomarkers, such as the use of phosphorylated tau protein in blood or cerebrospinal fluid and tau PET imaging and other tau protein-related indicators to track the progress of the disease. Some studies further show that specific plasma p-tau subtypes (including p-tau217) are elevated in the early stages of AD and continue to increase with the progression of the disease.

In animal studies, high-intensity intermittent training (HIIT) improved AD pathology by regulating the polarization of water channel protein-4 (AQP4) associated with astrocytic phenotypes (Feng et al., 2023). Yang et al. conducted long-term exercise pretreatment of TgF344-AD genetically modified rats and found that even after stopping exercise in the later stage of life, early and long-term exercise can improve the learning and memory ability of elderly rats and reduce anxiety or depression-like behavior. These behavioral benefits are accompanied by a reduction in Aβ deposition and excessive phosphorylation of tau protein, the reduction of neuronal damage, apoptosis and degeneration and the normalization of mitochondrial dynamics, while inhibiting oxidative stress and neuroinflammation (Yang et al., 2022). In an animal experiment by Belarbi and others, the wheel movement selectively reduced the pathological tau protein and there was no significant difference between the soluble pThr181-tau and the total tau protein, while the pathological tau protein signal was reduced. 33.4 ± 8.5% (*p* = 0.0373). The abnormal phosphorylation of the pSer422 site was also reduced, and these changes were not caused by the decrease in the expression of transgenic tau protein (Belarbi et al., 2011). In summary, exercise training may effectively reduce pathological tau protein, thus delaying the onset of AD and slowing down the progression of the disease.

## 3 Neuroinflammation and AD

In addition to the Aβ and tau protein hypothesis, neuroinflammation also plays a crucial role in the occurrence and development of AD. Broadly speaking, neuroinflammation refers to a local immune response in the central nervous system caused by factors such as protein aggregation, metabolism or vascular damage (Long et al., 2025). Immune microenvironment includes microglia, astrocytes, complement system, cytokine/chemokine network, and oxidative stress, which affecting synaptic function and neuronal survival (Zhang et al., 2024; Chu et al., 2026). Many studies show that neuroinflammation is an povital factor in the pathogenesis of AD. At present, the cascading reaction of neuroinflammatory mediated by activated glial cells or other immune cells in the brain is increasingly regarded as an important driving factor in the AD process (Thakur et al., 2023; Guo and Jiang, 2023). Microglia are the brain’s resident macrophages. In their resting state, they patrol the parenchyma. When triggered by AD-related signals, they activate, migrate, and clear Aβ while presenting antigens. But activation cuts both ways: it removes plaques in the short term, yet persistent activation drives chronic neuroinflammation—through cytokine release—that worsens neuronal injury (Merighi et al., 2022).

In AD, Aβ and tau function as both pathological deposits and immune activators—they stimulate glial cells and fuel neuroinflammation. Aβ, in particular, drives this pro-inflammatory program by upregulating NF-κB-dependent transcription of inflammatory genes and activating the complement cascade, which in turn orchestrates glial chemotaxis, phagocytosis, and mediator secretion (Zhao et al., 2023). Inflammatory signals further interact with tau protein pathology. For example, pathways such as p38 MAPK and Akt/mTOR are involved in the integration of inflammatory signals and lead to synaptic damage, while the activation of mTOR can inhibit autophagy and promote tau protein phosphorylation (Xue et al., 2024; Schnöder et al., 2020). In addition, the activation of NLRP3 inflammatory bodies mediated by the purine receptor P2X7R can enhance NF-κB signal transduction and may affect the permeability of the blood–brain barrier.

Targeted neuroinflammation has become a key strategy for preventing and treating AD. As a changeable lifestyle factor, exercise can reduce systemic inflammation, improve insulin resistance and vascular function, and reduce the continuous stimulation of peripheral inflammatory signals to the central nervous system (Pedersen and Toft, 2000). Animal studies show that continuous endurance training can downregulate the expression of inflammatory-related genes such as COX-2 and iNOS, significantly inhibit NF-κB activity, and reduce the levels of pro-inflammatory cytokines including TNF-*α*, IL-6 and IL-1β, while reducing tau protein phosphorylation and inflammation markers (Leem et al., 2011). In addition, short-term exercise can turn the immune response to adaptive immunity, which is related to the reduction of soluble Aβ, IL-1β and TNF-α levels in the hippocampus, indicating that exercise plays a role in breaking the vicious circle between Aβ and inflammation (Nichol et al., 2008). Vitamin D supplement combined with exercise (swimming for 30 min a day for 4 weeks) can significantly reduce the levels of IL-6, MDA, Aβ and tau protein, while significantly increasing IL-10, dopamine, BDNF and NGF levels (Medhat et al., 2020). Feng et al. reported that in the AD-like model (STZ-AD-like model) induced by streptomyceria of HIIT, HIIT promotes the polarization of glial cells and maintains the polarity of water channel AQP4, thus enhancing the lymphatic-renal clearance function (Feng et al., 2023). In human studies, although acute movement temporarily increases IL-6, it will then induce the production of anti-inflammatory mediators such as IL-1RA and IL-10, inhibit IL-1β signal conduction, and reduce inflammation-promoting factors such as TNF-α (Pinho et al., 2010).

Overall, exercise may improve neuroinflammation in AD through four ways. (A) Exercise inhibits the pro-inflammatory M1 phenotype and promotes the anti-inflammatory M2 phenotype, so as to maintain the immune homeosis. (B) Exercise upregulators neurotrophic factors such as BDNF and enhances the development of hippomatic nerves, thus promoting the generation of new neurons and glial cells and supporting tissue repair. (C) Exercise reduces the level of inflammatory mediators and weakens the activation of peripheral immune cells, thus limiting chronic inflammation. (D) Exercise repairs brain microvascular endothelium and closely connected damage, restores the permeability and integrity of the BBB, and reduces the entry of inflammatory mediators and immune cells into the brain (Pahlavani, 2023). Through these processes, exercise can inhibit neuroinflammatory reactions, delay the onset of AD, and alleviate symptoms.

## 4 Metabolism and AD

### 4.1 Glucose metabolism

Under physiological conditions, glucose is the main energy substrate of the brain, and ATP is produced by high-efficiency metabolism through glycolysis, the tricarboxylic acid cycle and oxidative phosphorylation. In the pathological process of AD, cerebral glucose metabolism disorder is considered an early event, usually occurring before Aβ plaque deposition (An et al., 2018). More and more evidence shows that impaired glucose uptake across the BBB has been regarded as an important biomarker associated with the development of AD, and the magnitude of cerebral hypometabolism is significantly correlated with the severity of cognitive decline (Butterfield and Boyd-Kimball, 2018). At the molecular level, insufficient expression of GLUT1 in BBB endothelial cells and glial cells, together with reduced neuronal GLUT3 on neuronal membranes, has been linked to more pronounced neurodegeneration and Aβ deposition, which may in turn accelerate cognitive deterioration (Winkler et al., 2015). Brain insulin resistance is a common pathological feature of AD. Under physiological conditions, insulin binds to neuronal insulin receptors to regulate the growth, survival, and synaptic plasticity of neurons by phosphorylating IRS-1 and activating the PI3K/Akt pathway (Bomfim et al., 2012). In AD, impaired insulin signaling and reduced IRS-1 phosphorylation may stem from aberrant JNK activation. This JNK dysregulation then alters the transcriptional program of glucose-metabolism genes, compromising neuronal glucose utilization and ultimately driving memory deficits (Yarza et al., 2015).

Based on the pathogenic mechanisms outlined above, targeted improvement of cerebral energy metabolism and insulin sensitivity has emerged as an important strategy for AD intervention. In animal studies, long-term, regular aerobic exercise not only improves cognitive performance in APP/PS1 transgenic AD mice but also selectively promotes glucose metabolic reprogramming of hippocampal microglia and enhances their morphological plasticity (Zhang et al., 2022). Similarly, Liang et al. subjected 3-month-old APP/PS1 mice to 12 weeks of treadmill training and found that exercise improved Morris water maze performance, reduced hippocampal Aβ40/42 burden, and suppressed the NLRP3 inflammasome. Mechanistically, exercise mitigated inflammation and AD-like pathology by reprogramming microglial glucose metabolism, characterized by enhanced glycolysis and oxidative phosphorylation (OXPHOS) and an increased oxygen consumption rate/extracellular acidification rate (OCR/ECAR) ratio (Liang et al., 2025). Collectively, exercise may delay AD progression and alleviate symptoms through modulation of glucose metabolism.

### 4.2 Lactate metabolism

Lactate has long been regarded as a metabolic by-product or even a waste. However, more recent studies indicate that lactate in the brain can serve as an energy substrate (Brooks, 2020). Cerebral lactate is derived mainly from glycolysis and glycogenolysis in astrocytes and can be shuttled between glial cells and neurons. Notably, even under normoxic conditions, astrocytes and other highly active cells can generate lactate through aerobic glycolysis, a phenomenon commonly referred to as the Warburg effect (Yellen, 2018). Under physiological conditions, lactate may contribute approximately 10% to cerebral energy metabolism, whereas its contribution can rise to ~60% when lactate availability is increased (Boumezbeur et al., 2010). Clinical studies frequently observe elevated CSF lactate in AD compared with healthy controls, and CSF lactate may be higher in early-stage disease than in more advanced stages (Zebhauser et al., 2022). One possible interpretation is that mitochondrial metabolism is disrupted in early AD, prompting a compensatory shift toward glycolytic flux, whereas glycolytic metabolism may deteriorate as neurodegeneration progresses.

Using a mouse model with AD, Han and his colleagues found that treadmill exercise can reduce cognitive disorders and synaptic loss. It is worth noting that systemic injection of sodium lactate can also reproduce the beneficial effects of exercise, which supports the view that lactic acid may be a key mediator for these neuroprotective effects (Han et al., 2025). In a randomized trial, Green et al. found that acute cycling decreased gray matter FDG uptake in 60 older adults—an effect tied to both lactate production and workout intensity. The motor cortex was the lone exception, showing increased uptake. These findings imply a transient brain-wide fuel shift from glucose to lactate, except in motor cortex (Green et al., 2026).

### 4.3 Mitochondrial dysfunction

Mitochondria are highly complex organelles that play a central role in cell energy production, metabolic regulation, calcium homeostasis, ROS generation, and cell apoptosis control (Chan, 2006). In addition to providing energy, mitochondria also maintain dynamic balance through continuous fusion and division, which is crucial to maintaining organelle integrity and ensuring their correct position and distribution in neuronal projections. Similar to glucose metabolic disorders, mitochondrial dysfunction occurs in the early stage of AD and forms a vicious circle with Aβ accumulation and oxidative stress (Wang et al., 2022). Aβ-induced oxidative stress can trigger mitochondrial division procedures, such as the Drp1/Fis1 pathway, causing excessive fragmentation of mitochondria, hindering their transport to synapses, and reducing the availability of local ATP, which ultimately leads to synaptic dysfunction (Reddy and Oliver, 2019). In addition, both Aβ and phosphorylated tau protein can interact with Drp1 and enhance its activity, thus further aggravating mitochondrial kinetic abnormalities and ensuing synaptic defects.

Appropriate exercise is beneficial to mitochondrial autophagy and mitochondrial function, which can promote mitochondrial plasticity, reduce oxidative stress, improve cognitive ability, and reduce the risk of cognitive disorders and dementia (Liang et al., 2021). Studies on the hippocal body show that exercise training can reduce mitochondrial fragmentation, down-regulated expression of Drp1 and Fis1, and increase the level of fusion-related proteins (such as OPA1, MFN1 and MFN2), thus improving mitochondrial morphology and function (Li et al., 2019). Cai et al. reported that in the 5 × FAD mouse model, aerobic exercise promotes the transfer of “healthy mitochondria” from astrocytes to neurons by upregulating glial CD38, thus improving mitochondrial quality, reducing oxidative stress, and reducing A*β* pathology and cognitive dysfunction. It is worth noting that CD38 knocking down can damage mitochondrial transfer and eliminate these benefits from exercise (Cai et al., 2025). In the seahorse of elderly rats, treadmill exercise enhanced PINK1-Parkin-dependent mitochondrial autophagy, improved resistance to age-related mitochondrial dysfunction, and alleviated Aβ-induced cognitive decline and mitochondrial injury in AD animal models (Zhao et al., 2021). In summary, restoring mitochondrial function through exercise may delay the onset of AD and slow down the progression of the disease.

### 4.4 Lipid metabolism

The brain is highly enriched in lipids, which not only constitute essential structural components of synaptic and myelin membranes but also participate broadly in cellular signaling and can serve as energy substrates (Naudí et al., 2015). Because cholesterol metabolism in the brain is largely regulated autonomously within the central nervous system, intercellular lipid trafficking is essential for maintaining the composition and function of neuronal membranes (Vance et al., 2005). In particular, astrocytes play a key role in transporting cholesterol and other lipids to neurons through lipoprotein particles containing apolipoprotein E (ApoE) (Liu et al., 2013). It is worth noting that the ApoE4 isomer shows a decrease in lipid binding ability and a decrease in secretion and transport efficiency, which makes the brain more prone to lipid homeostasis.

In terms of mechanism, lipid metabolism disorders can aggravate the core pathological changes of AD in many ways. The amyloid production process of APP mainly occurs in the fat raft microdomains rich in cholesterol and sheath, and is mediated by β-secreting enzymes and *γ*-secreting enzymes (Hur, 2022). Increased cholesterol levels increase the activity of these secretory enzymes, thus promoting the production of Aβ (Hur, 2022). Specifically, extracellular palmitic acid induces the expression of BACE1 and APP, thereby promoting Aβ production in SK-N-MC cells through the Akt-NF-κB and Akt–mTOR-HIF-1α pathways (Kim et al., 2017). Twelve weeks of dietary docosahexaenoic acid supplementation reduced Aβ accumulation in the brain by upregulating insulin-degrading enzyme (IDE) expression (Hashimoto et al., 2008). In animal models, inhibiting cholesterol synthesis in astrocytes can reduce the cholesterol content of neurons, make APP turn to the non-amyloid production pathway, and accompanied by the reduction of pathological changes in Aβ and tau proteins (Wang et al., 2021). In addition, the ceramides produced by the hydrolysis of sheath phospholipids can promote the production of amyloids, and the oxidative stress induced by Aβ will further activate sheath phospholipase, forming a positive feedback loop that links lipid disorders with Aβ deposition (Xing et al., 2023; Czubowicz et al., 2019). In addition to pathological changes in amyloid protein, lipid imbalance may also promote excessive phosphorylation of tau protein by aggravating neuroinflammation and activating signaling pathways such as PI3K–Akt/GSK3β/Cdk5 (Yang et al., 2020). In addition, oxidized sterols such as 27-hydroxy cholesterol can upregulate the expression of BACE1 by activating the NF-κB signaling pathway, thus mechanically linking lipid imbalance with an increase in Aβ production (Wu et al., 2022). RXR agonists enhanced Aβ clearance and improved cognitive deficits in AD mouse models by upregulating ABCA1 activity (Boehm-Cagan and Michaelson, 2014). In short, these mechanisms show that cerebral lipid metabolism disorder is a pathological node that can be intervened in AD. In line with this view, the cognitive benefits of improving interventional AD risk factors through lifestyle interventions (such as physical exercise and diet adjustment) seem to be related to the ApoE genotype to some extent and is usually more significant in non-ApoE4 carriers (Liu et al., 2013).

### 4.5 Brain-derived neurotrophic factor

Brain-derived neurotrophic factor (BDNF) is a key protein that supports the survival, growth, and synaptic plasticity of adult brain neurons. It is mainly synthesized in the cytobody of neurons and glial cells, and then reaches the presynaptic end and dendritic area through axon transport. In glutamic acid energy synapses, BDNF binds to its high-affinity receptor TrkB to regulate neurotransmitter release, ion channel activity, axon guidance and neuronal excitability, so it is crucial to learning and memory formation (Schinder and Poo, 2000). More and more evidence shows that the decrease in BDNF levels is associated with a variety of pathological processes of AD, including Aβ deposition, excessive phosphorylation of tau protein, neuroinflammation and neuronal apoptosis (Jiao et al., 2016; Nagahara et al., 2013). In APP/PS1 transgenic AD mice, exogenous BDNF reduces the production of toxic Aβ by promoting alpha-secretase enzyme-mediated APP processing, indicating that BDNF can directly regulate the metabolic pathway of amyloid protein (Nigam et al., 2017).

Evidence showed exercise-induced BDNF decreases the generation of neurotoxic Aβ peptides by enhancing *α*-secretase cleavage of APP (Nigam et al., 2017). It is worth noting that the expression of BDNF can also be regulated by external intervention. Choi and his colleagues conducted an animal study of 5 × FAD transgenic mice, which showed that exercise can improve cognitive function while inducing adult hippocampal nerve growth and increasing the level of hippocampal BDNF. The author further analyzed a variety of hippoma molecular markers and found that the movement simultaneously upregulated the expression of BDNF and synaptic-related proteins PSD95 and SYP, accompanied by an increase in FNDC5 levels. In contrast, the intervention of only increasing AHN failed to reproduce these molecular changes (Choi et al., 2018). Belaya et al. conducted long-term voluntary rotation experiments on 5 × FAD mice and found that compared with the sedentary control group, exercise increased the total level of hippocampal BDNF by about 18% (Belaya et al., 2020). Previous studies have shown that aerobic exercise can significantly increase the plasma BDNF level of patients with AD, and the level of BDNF is significantly related to the level of physical activity (Coelho et al., 2014). In addition, Valenzuela et al. reported that regular exercise can enhance the production of neurotrophic factors (such as BDNF) by increasing metabolic factors such as ketones and lactic acid, as well as muscle-derived myocytokines, including histoproteinase B and iris (Valenzuela et al., 2020). In general, exercise not only directly or indirectly adjusts neurotrophic factors such as BDNF, but also improves the accompanying pathological state, such as oxidative stress, thus exerting a multi-target, systematic neuroprotective effect and reducing the cognitive and non-cognitive disorders of AD (Belviranlı and Okudan, 2019).

### 4.6 Iron metabolism

In the pathological progression of Alzheimer’s disease, a large body of evidence indicates that abnormal accumulation of redox-active iron (Fe^2+^) and disruption of iron homeostasis can drive the progressive decline of cognitive function by inducing oxidative stress, promoting aberrant protein aggregation, and triggering iron-dependent cell damage (ferroptosis) (Li et al., 2020). Studies have shown that the expression of ferritin and divalent metal transporter 1 (DMT1) is upregulated in neurons of AD mice, while transferrin receptor 1 (TfR1) and ferroportin 1 (Fpn1) are decreased (Lu et al., 2017). Chronic treadmill training for eight weeks attenuated Aβ-induced neuronal cell death and cognitive decline via promotion of *α*-secretase-dependent APP processing, which was mediated by low iron-induced augmentation of furin activity (Choi et al., 2021). These findings indicate that exercise suppresses neuronal iron overload and facilitates neuronal iron efflux, thereby mitigating iron accumulation and diminishing brain iron deposits.

## 5 Conclusion

Alzheimer’s disease represents a multifactorial neurodegenerative disorder driven by the intricate crosstalk between canonical protein pathology, chronic neuroinflammation, and cerebral metabolic dysfunction, rather than isolated pathological alterations. Accumulating evidence consistently demonstrates that physical exercise serves as a potent, multi-targeted neuroprotective strategy to counteract AD progression through the modulation of multiple core pathogenic pathways. In summary, exercise effectively mitigates AD-related proteinopathy by inhibiting aberrant Aβ aggregation and deposition as well as pathological tau hyperphosphorylation and neurofibrillary tangle formation. Furthermore, exercise suppresses excessive neuroinflammation by restraining microglial and astrocytic overactivation, thereby interrupting the pro-inflammatory positive feedback loop that exacerbates neuronal injury. Critically, exercise remodels cerebral metabolic homeostasis comprehensively: it enhances cerebral glucose uptake and insulin sensitivity, optimizes brain lactate metabolism and energy shuttling, preserves mitochondrial structural integrity and functional activity to alleviate oxidative stress, and ameliorates disordered cerebral lipid metabolism. As a pivotal upstream neurotrophic regulator, exercise-induced BDNF upregulation further improves cognitive function in AD.

Notably, compared with conventional pharmacological therapies that often target a single pathological molecule or signaling pathway, physical exercise exhibits unique superiority in intervening the complex interactive pathological network of AD, covering protein metabolism, inflammatory response, energy metabolism, and neurotrophic regulation. Nevertheless, several limitations and unresolved issues remain in current research. The optimal exercise modality, intensity, duration, and intervention window for different AD pathological stages have not been standardized. In addition, the precise molecular mechanisms underlying exercise-mediated crosstalk between metabolic remodeling, neuroinflammatory regulation, and Aβ/tau clearance, as well as the specific upstream and downstream signaling of BDNF in this regulatory network, require further in-depth validation. Most existing studies also lack long-term clinical longitudinal data and individual mechanism verification, restricting the precise clinical transformation and application of exercise intervention strategies. Practically, for individuals with dementia, safety and efficacy should be prioritized through pre-exercise screening, individualized program design, and professional supervision to prevent adverse events. Participants should undergo pre-exercise medical screening, and programs should be individually tailored to accommodate physical comorbidities, mobility limitations, and cognitive status.

Future research should focus on clarifying the stage-specific neuroprotective effects of different exercise paradigms, exploring the key molecular nodes connecting exercise-induced metabolic reprogramming and pathological improvement, and validating the BDNF-centered synergistic regulatory network in human clinical samples. Elucidating these mechanisms will help establish standardized, personalized exercise intervention protocols for AD prevention and treatment. Collectively, this review confirms that physical exercise is a reliable non-pharmacological approach for AD intervention, and further exploration of its multi-target neuroprotective mechanisms will provide novel insights and actionable strategies for delaying and treating Alzheimer’s disease.
